# Modeling of Dynamic Recrystallization Evolution for Cr8 Alloy Steel and Its Application in FEM

**DOI:** 10.3390/ma15196830

**Published:** 2022-10-01

**Authors:** Xuewen Chen, Bingqi Liu, Bo Zhang, Jiawei Sun, Zhen Yang, Xudong Zhou, Tao Huang, Danqing Yin

**Affiliations:** School of Materials Science and Engineering, Henan University of Science and Technology, 263 Kaiyuan Avnue, Luyang 471023, China

**Keywords:** Cr8 alloy steel, dynamic recrystallization, finite element method, high temperature compression test, microstructure evolution

## Abstract

In the process of Cr8 roller production, the phenomenon of coarse grain size and uneven grain size often appears, which makes the mechanical properties of the material decrease sharply. Accurate dynamic recrystallization model is the basis for predicting the change of grain size during thermal processing, and is an important basis for refining grain and improving material properties. In this study, the isothermal compression experiment was carried out on Cr8 alloy steel at 900–1200 °C and 0.005–0.1 s^−1^ by Gleeble –1500D thermal simulation compressor, and the stress dates of Cr8 alloy steel were obtained. According to experimental data, the Kopp dynamic recrystallization model of Cr8 alloy steel was established. The dynamic recrystallization volume fraction obtained by Kopp model was compared with that obtained by experiment at the same temperature and strain rate. The correlation value was 0.988, and the root mean square error (RMSE) was 0.053, which proved that the DRX model established was reliable. Through the secondary development of the program, the DRX model of Cr8 alloy steel was written into the software Forge^®^ to verify the microstructure evolution model. The compression process of a cylindrical specimen of Cr8 alloy steel at 0.1 s^−1^ and 1050 °C was simulated, and the DRX microstructure evolution of the alloy was calculated. The comparison between the final grain size calculation results and the test metallographic photos of samples in different deformation zones shows the relative error of the grain size was less than 10.6%, indicating that the DRX model of Cr8 alloy steel can better predict the dynamic recrystallization of Cr8 alloy steel.

## 1. Introduction

As key components of rolling mills, rollers need to have good mechanical properties. When the microstructure of the roller has coarse grains and mixed grains, the mechanical properties of the roller will decrease, the service conditions of the rolling mill cannot be met, and the service life will be reduced. Cr8 alloy steel, as a new type of cold rolled steel, will experience dynamic recrystallization (DRX) behavior during the hot forming process of the roller, and the dynamic recrystallization behavior of metals is a key factor affecting the evolution of the microstructure. After the metal undergoes DRX, the coarse grains are often replaced by new fine grains, which promotes grain refinement and enables the metal to have better mechanical properties. However, in the process of metal rolling, the dynamic recrystallization volume fraction of each deformed region is different due to the different deformation degree, which makes it difficult to determine the grain size of different regions, and the rolling product cannot be guaranteed to have excellent mechanical properties. Therefore, it is necessary to establish an efficient method to predict the dynamic recrystallization volume fraction and grain size of material, and then optimize the forming process of the roller and improve the strength and wear resistance of Cr8 alloy steel. In this regard, it is important to study the dynamic recrystallization behavior and grain evolution characteristics of Cr8 alloy steel.

The dynamic recrystallization behavior has an important effect on the microstructure evolution and grain size of materials; therefore, the dynamic recrystallization behavior and mechanism of metals have been analyzed by numerous researchers. Jiachen Li et al. [1] introduced the Z parameter to analyze the DRX mechanism of Al–Mg–Si alloy under different ln Z. Jingjing Zhang et al. [2] discussed the recrystallization mechanism of 2195 aluminum alloy at different temperatures. It was mainly high temperature continuous dynamic recrystallization and medium temperature discontinuous dynamic recrystallization, indicating that temperature affects the recrystallization mechanism of aluminum alloy. Zhi Jia et al. [3] found that when the temperature reached above 1100 °C, the DRX mechanism of Inconel 625 alloy was mainly continuous dynamic recrystallization (CDRX). M. Leonard et al. [4] studied the significant change of DRX mechanism of Zn–Cu–Ti alloy caused by strain rate through the tensile test of Zn–Cu–Ti plate. H.T. Jeong et al. [5] researched the recrystallization mechanism of high−entropy alloy and determined that the DRX mechanism of the high−entropy alloy was inconsistent under different conditions. Xinyun Wang et al. [6] found when the strain rate was higher than 0.1 s^−1^, the DRX mechanism of austenitic stainless steel was CDRX, and when the strain rate was less than 0.1 s^−1^, the DRX mechanism of austenitic stainless steel was DDRX.

While studying the dynamic recrystallization mechanism of a large number of alloys, many scholars have also carried out research to quantitatively characterize the influences of deformation feature (temperature and strain et al.) on the dynamic recrystallization (DRX) volume fraction of alloys. Sellars [7,8], Yada [9], Kim [10,11], H.J. McQueen [12], Kopp [13,14] et al. studied different materials and proposed different microstructure evolution models. The DRX volume fraction model proposed by Yada and Kopp has clear parameters and was easy to determine, which was widely used [15,16,17]. By establishing the DRX model of materials, a new idea was provided for characterizing the dynamic recrystallization behavior of metals, and a theoretical basis was provided for formulating the grain refinement process. Fei Chen et al. [18] built the DRX model of 30Cr2Ni4MoV steel, through which the deformation behavior and the evolution of recrystallization structure of the metal was characterized. Mohd Kaswandee Razali et al. [19] fitted the dynamic recrystallization behavior of medium manganese steel by establishing the rheological stress model and Avrami dynamic model of medium manganese steel, predicted its microstructure evolution, and avoided the solution of characteristic strain. Bin Fang et al. [20] built the DRX model of FGH96 P/M alloy to predict the microstructure of this metal. These scholars have established DRX models of different materials, which were of great significance for optimizing the microstructure of materials. Although the dynamic recrystallization model of many materials has been established by scholars, it is helpful to study the dynamic recrystallization behavior of materials. However, they only establish the dynamic recrystallization model of the material, and qualitatively analyze the microstructure evolution inside the material through metallographic photographs. There is no other effective method to quantitatively predict the microstructure evolution of materials in different regions during thermal deformation. Therefore, some scholars try to combine the dynamic recrystallization model with the finite element method, and embed the established dynamic recrystallization model into the finite element software to predict the microstructure evolution of the material. He Jiang et al. [21] simulated the microscopic evolution of 690 alloy used simulation software combined with DRX model, and analyzed the DRX process of 690 alloy. R. A. R. Giorjao et al. [22] simulated the compression process of AZ31B alloy cylindrical sample and predicted the grain size of the alloy during compression. Hongchao Ji et al. [23] built the recrystallization model of 33Cr23Ni8Mn3N heat resistant steel and predicted the dynamic recrystallization behavior of deformed metal. However, there are few studies on predicting the microstructure evolution of Cr8 alloy steel based on the dynamic recrystallization model embedded in finite element software. Therefore, it is necessary to study the dynamic recrystallization behavior of Cr8 alloy steel and its microstructure evolution during hot forming based on the dynamic recrystallization model of Cr8 alloy steel and finite element software.

In order to effectively predict and control the grain size of the metal during the hot forming process, this paper uses the Gleeble−1500D thermal simulation compression tester to conduct the hot compression test of Cr8 alloy steel at 900–1200 °C and 0.005–0.1 s^−1^. The flow stress curves of Cr8 alloy steel at different temperatures and strain rates were obtained. Based on the true stress−strain curve of Cr8 alloy steel and the Kopp dynamic recrystallization model, the dynamic recrystallization model of Cr8 alloy steel was established and embedded in the forming finite element software Forge^®^. Based on the dynamic recrystallization model established in this paper, taking the strain rate of 0.1 s^−1^ as an example, a cylindrical compression test of Cr8 alloy steel was simulated. The average grain size obtained from the simulation was compared with the grain size obtained from the metallographic photographs to verify the accuracy of the model, provide data and technical support for optimizing the thermal processing parameters of Cr8 alloy steel roll and effectively control its internal microstructure.

## 2. Experiment Method

**Figure 1:**
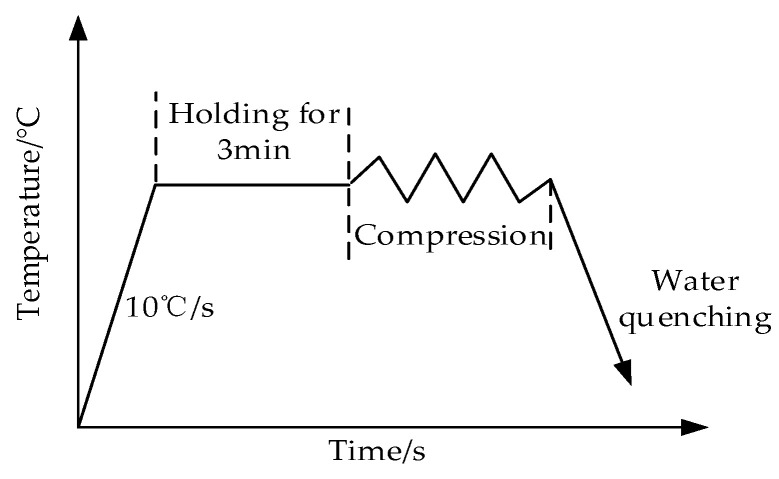
Experimental processing of Cr8 alloy steel.

The metal studied in this paper was Cr8 alloy steel for large cold roller, and the chemical composition was recorded in Table 1. Cylindrical sample with a size of ø 8 × 12 mm was used. Compression test of metal was achieved on Gleeble–1500D Thermomechanical Simulation Tester (Dynamic Systems Inc, New York, NY, USA). The details of the experimental method are shown in Figure 1. In order to make the stress of the sample uniform during deformation and reduce the influence of friction, we evenly apply water–graphite lubricant on both ends of the sample, and heat the sample to the experimental temperature (900 °C, 975 °C, 1050 °C, 1125 °C and 1200 °C) at the speed of 10 °C/s, and then keep the temperature for 3 min [16,24] to make the internal and external temperature of the sample uniform. The compression test was executed at 0.005 s^−1^, 0.01 s^−1^ and 0.1 s^−1^, and the compressive strain was 0.65. The strain rate is calculated by the equation: $\dot{\varepsilon }=d\varepsilon /dt$, and controlled by computer closed−loop to realize constant strain rate test. Because the deformation of the sample in this experiment is not large and the bulging effect is not obvious, there is no correction. After the deformation, the sample was put into water for cooling immediately to retain the microstructure of metal after high–temperature deformation to the greatest extent. After polishing, the compressed sample was corroded with a mixed solution of 5 g of picric acid, 2 g of dodecyl phenol and 100 mL of distilled water, and the micrograph of the sample was obtained by Olympus–pmg3 (Olympus Corporation, Tokyo, Japan) metallurgical microscope.

## 3. Results and Analysis

### 3.1. Flow Stress Curve of Cr8 Alloy Steel

**Figure 2:**
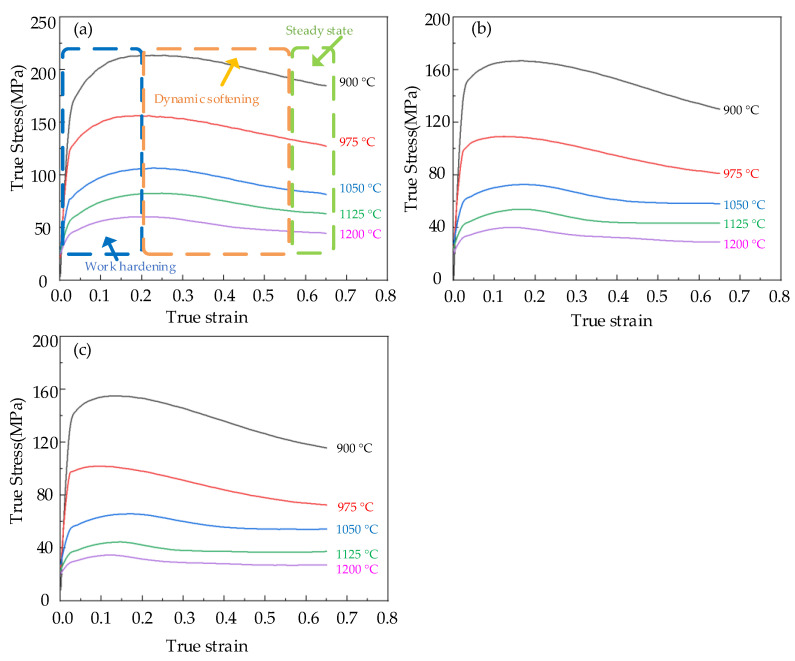
True Stress Strain Curve of Cr8 Alloy Steel. Under different conditions: (**a**) $\dot{\varepsilon }$ = 0.1 s^−^^1^; (**b**) $\dot{\varepsilon }$ = 0.01 s^−^^1^; (**c**) $\dot{\varepsilon }$ = 0.005 s^−^^1^.

The high temperature compression test of Cr8 alloy steel was implemented by Gleeble–1500D Thermomechanical Simulation Tester, and the true stress−strain curve of Cr8 alloy steel was obtained directly by Gleeble–1500D Thermomechanical Simulation Tester and recorded in Figure 2. It can be seen from Figure 2 that the flow stress curve of Cr8 alloy steel is a typical DRX curve. At the beginning of specimen compression, the stress value of metal increases rapidly because of the accumulation of dislocations. When the critical stress was reached, new grains began to appear inside the material, and dynamic recrystallization occurred, which promoted the rising trend of stress to slow down. At this stage, the work hardening of the material plays a leading role, increasing the stress and reaching the peak stress; in the dynamic softening stage, as new grains nucleated and grew, dynamic recrystallization (DRX) and dynamic recovery (DRV) begun to predominate. In the steady state stage, the stress value decreases continuously with increasing strain, and the DRV, DRX, and work hardening tend to balance eventually. As described in Figure 2a, at 0.1 s^−1^ the metal first achieved the peak stress $\sigma_{p}$; the peak stress at 900–1200 °C was 213.72 MPa, 156.42 MPa, 106.63 MPa, 82.66 MPa, 60.33 MPa. As the metal continued deforming, the dynamic recovery and recrystallization inside the material continued to occur, which reduced the stress value and finally reached the steady–state stress value $\sigma_{\mathrm{ss}}$, and the steady–state stress at 900–1200 °C was 184.24 MPa, 123.63 MPa, 82.42 MPa, 64.24 MPa, 46.06MPa. The values of steady state stress and peak stress of Cr8 alloy steel at 900–1200 °C and 0.005–0.1 s^−1^ are recorded in Table 2. Table 2 shows that with the decrease of strain rate, the values of $\sigma_{p}$ and $\sigma_{\mathrm{ss}}$ of Cr8 alloy steel also decreased. For example, at 900 °C, when the strain rate of Cr8 alloy steel was reduced from 0.1 s^−1^ to 0.005 s^−1^, the peak stress changed from 213.72 MPa to 155.46 MPa, and the steady state stress decreased from 184.24 MPa to 108.08 MPa.

### 3.2. Establishment of Recrystallization Model of Cr8 Alloy Steel

#### 3.2.1. Identification Parameters of Critical Strain Model

Dynamic recrystallization was the process of forming new equiaxed crystals inside Cr8 alloy steel. The occurrence of the DRX required the accumulation of sufficient dislocation energy; therefore, the DRX could occur when a critical strain was reached. Poliak et.al. [21] found that when the material undergoes recrystallization, the work−hardening rate *θ* will have a turning point. The value of *θ* was calculated by

(1) θ=∂σ/∂ε

where σ is the stress, *θ* is the work−hardening rate and the ε is the strain.

The critical stress was the stress at the turning point, and the critical strain was the strain at the turning point. Subsequently, Equation (2) was derived as follows:

(2) $$\frac{\partial \theta }{\partial \sigma }=\frac{\partial \theta }{\partial \varepsilon }\times \frac{\partial \varepsilon }{\partial \sigma }=\frac{\partial \theta }{\partial \varepsilon }\times \frac{1}{\theta }=\frac{\partial \left(\ln\theta \right)}{\partial \varepsilon }.$$

Therefore, the values of critical strain can be solved by extreme value of the ∂(ln(θ))/∂ε−ε curve, and the relationship between ln(θ) and *ε* found in the literature can be represented by a cubic spline fitted curve, such as

(3) $$\ln\theta =A+B\varepsilon +C\varepsilon^{2}+D\varepsilon^{3}.$$

Therefore, Equation (4) was derived as

(4) $$\frac{\partial \left(\ln\theta \right)}{\partial \varepsilon }=B+2C\varepsilon +3D\varepsilon^{2}.$$

Therefore, the critical strain $\varepsilon_{c}$ can be obtained by

(5) $$\varepsilon_{c}=-\frac{C}{3D}.$$

**Figure 3:**
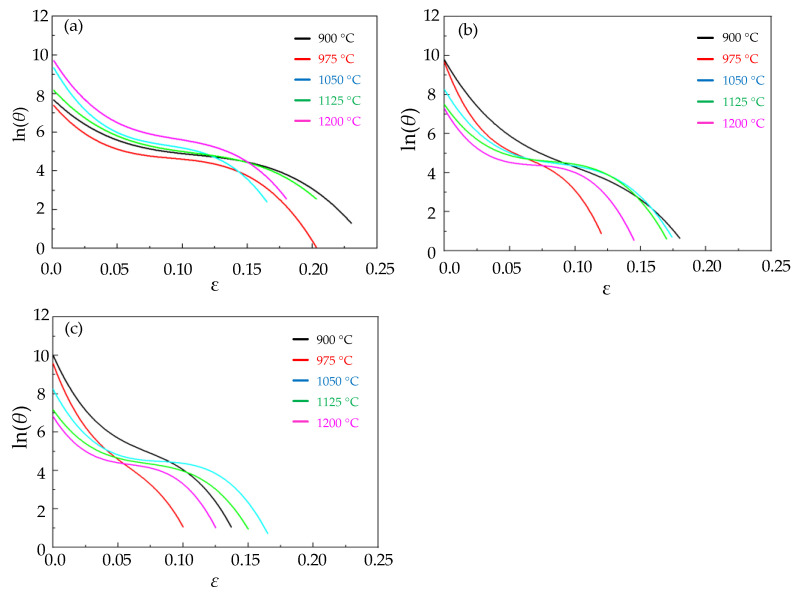
The relationship map of ln(θ)−ε under different temperatures and strain rates: (**a**) $\dot{\varepsilon }$ = 0.1 s^−^^1^; (**b**) $\dot{\varepsilon }$ = 0.01 s^−^^1^; (**c**) $\dot{\varepsilon }$ = 0.005 s^−^^1^.

**Figure 4:**
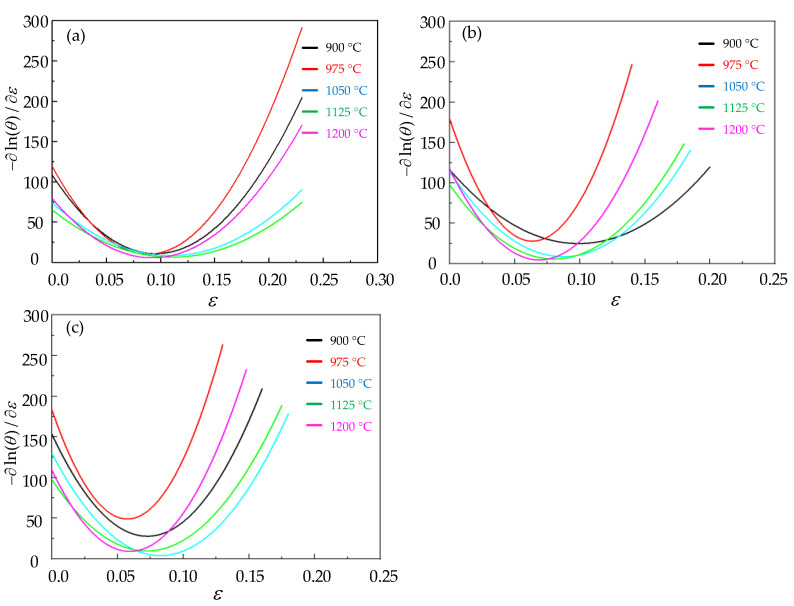
The relationship map of −∂(ln(θ))/∂ε−ε under different temperatures and strain rates: (**a**) $\dot{\varepsilon }$ = 0.1 s^−^^1^; (**b**) $\dot{\varepsilon }$ = 0.01 s^−^^1^; (**c**) $\dot{\varepsilon }$ = 0.005 s^−^^1^.

The fitted ln(θ) − *ε* curves of Cr8 alloy steel are depicted in Figure 3. All curves show the same trend. When the strain keeps increasing, ln(θ) gradually decreases and tends to a certain value and then decreases rapidly. Figure 4 is the curve diagram after being fitted by −∂(ln(θ))/∂ε − *ε*, showing that each curve has a minimum value corresponding to the critical strain of Cr8 alloy steel.

Through Figure 3 and Figure 4, the fitted parameters of ln(θ)−ε of Cr8 alloy steel under different conditions were obtained, as well as the cubic spline fitted curve of different conditions. The critical strain $\varepsilon_{c}$ under corresponding conditions was calculated by Formula (5). At 0.005 s^−1^ and 900 °C, the ln(θ)−ε fitted curve of Cr8 alloy steel was obtained as

(6) $$\ln\theta =10.001-152.961\times \varepsilon +1723.502\times \varepsilon^{2}-7913.370\times \varepsilon^{3}.$$

Taking 0.005 s^−1^ and 900 °C as an example, the critical strain of Cr8 alloy steel was 0.0726.

The fitted parameters, critical strains and peak strains are recorded in Table 3.

**Figure 5:**
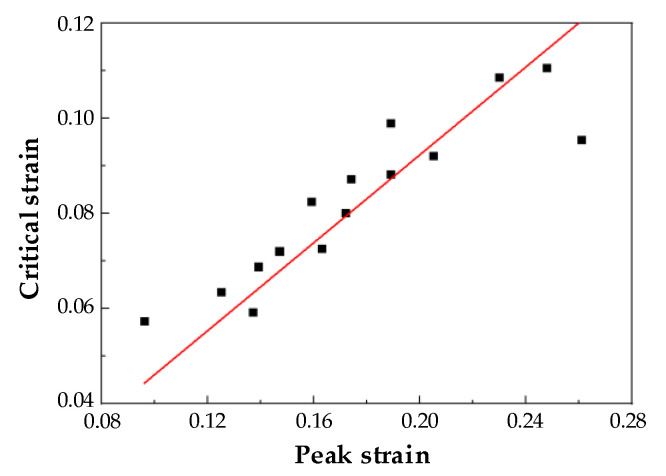
The relationship map of $\varepsilon_{p}$ and $\varepsilon_{c}$ under different temperatures and strain rates.

Performing linear regression on peak and critical strains in Table 3, as depicted in Figure 5, displays that $\varepsilon_{p}$ and $\varepsilon_{c}$ have a linear relationship: $\varepsilon_{c}$ = 0.46143$\varepsilon_{p}$.

The peak strain model of Cr8 alloy steel was calculated according to the literature, and the model Formula is depicted in

(7) $$\varepsilon_{p}=A_{p}\cdot d_{0}{}^{n_{p}}\cdot {\dot{\varepsilon }}^{m_{p}}\cdot \exp\left(\frac{Q_{p}}{RT}\right)$$

where $d_{0}$ represents the grain size before deformation, $\dot{\varepsilon }$ stands for strain rate, $Q_{P}$ is the critical strain activation energy, *R* is the gas constant, *R* = 8.3145 J/(mol K), T is the Kelvin temperature, and $A_{P}$, $n_{P}$, $m_{P}$ are constants.

The role of initial grain size was not investigated; therefore, the default initial grain sizes are all the same, and $d_{0}{}^{n_{p}}$ can be regarded as a constant. Equation (8) was obtained as

(8) $$\varepsilon_{p}=A_{p}\cdot {\dot{\varepsilon }}^{m_{p}}\cdot \exp\left(\frac{Q_{p}}{RT}\right).$$

Taking the logarithm of Equation (8) yields

(9) $$\ln\varepsilon_{p}=\ln A_{p}+m_{p}\ln\dot{\varepsilon }+\frac{Q_{p}}{RT}.$$

**Figure 6:**
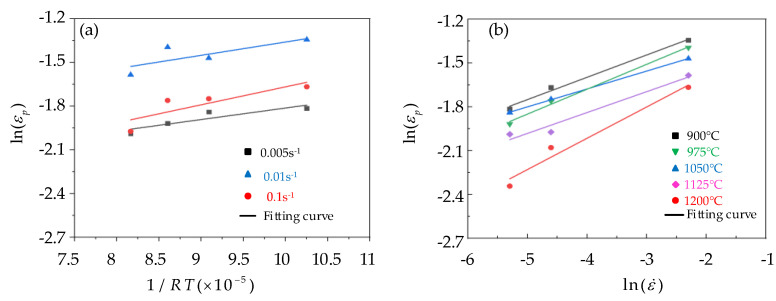
Determination of peak strain model parameters based on the control single variable method: (**a**) The relationship map of $\ln(\varepsilon_{p})-1/RT$; (**b**) The relationship map of $\ln(\varepsilon_{p})-\ln\dot{\varepsilon }$.

When the strain rate was constant, then $\ln(A_{p})+m_{p}\ln(\dot{\varepsilon })$ was a constant, and the scatter plot of $\ln(\varepsilon_{p})-1/RT$ was established and fitted, as depicted in Figure 6a, then the slope of the fitted curve $\ln(\varepsilon_{p})-1/RT$ was equal to the $Q_{P}$ value. According to Figure 6a, the $Q_{P}$ values under different conditions were obtained, and take its average value to obtain $Q_{P}$ = 9802.7955 J/mol. When the temperature was constant, $\ln(A_{p})+Q_{p}/RT$ was also a constant, and a scatter plot of $\ln(\varepsilon_{p})-\ln\dot{\varepsilon }$ at different temperatures was established, a linear fit was performed, as shown in Figure 6b, and $m_{P}$ = 0.160762 was calculated. Substitute the values of $m_{P}$ and $Q_{P}$ into Formula (7) to obtain the $A_{P}$ value under different conditions, and the average can be obtained as $A_{P}$ = 0.13497. Therefore, the peak strain model was

(10) $$\varepsilon_{p}=0.13497\cdot {\dot{\varepsilon }}^{0.160762}\cdot \exp\left(\frac{9802.7955}{RT}\right).$$

The critical strain model was

(11) $$\varepsilon_{c}=0.46143\varepsilon_{p}=0.0622792\cdot {\dot{\varepsilon }}^{0.160762}\cdot \exp\left(\frac{9802.7955}{RT}\right).$$

#### 3.2.2. Determination of Dynamic Recrystallization Model Parameters

**Figure 7:**
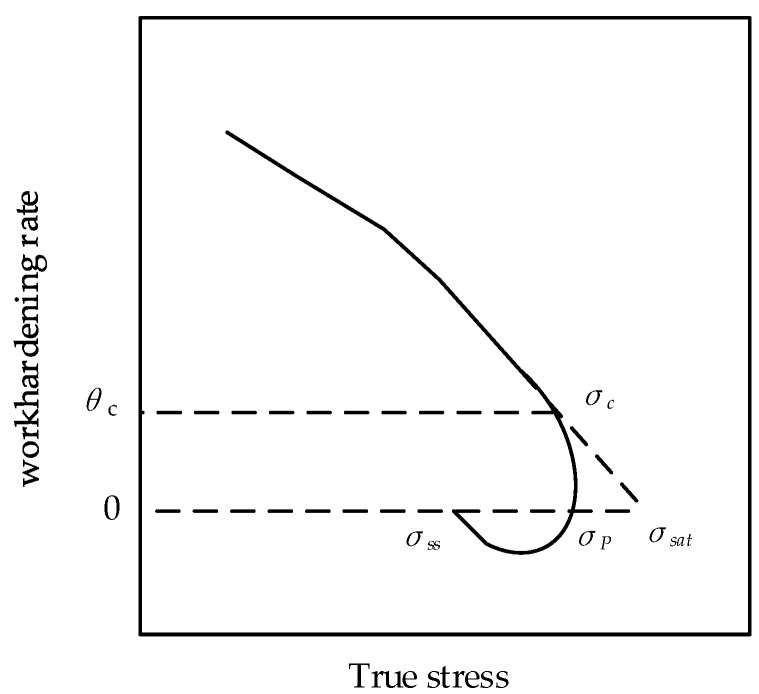
Typical relation curve of θ−σ.

**Figure 8:**
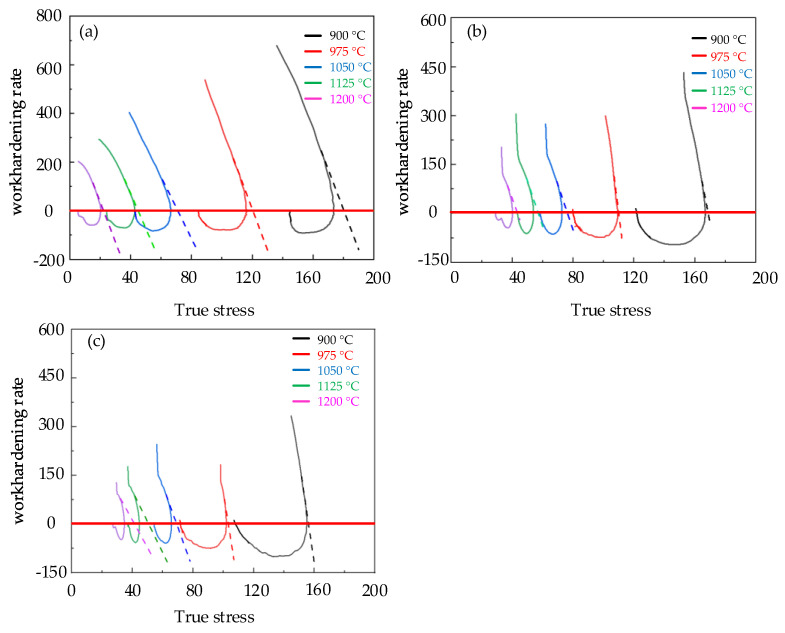
Relation curve of θ−σ of Cr8 steel under different temperatures and strain rates: (**a**) $\dot{\varepsilon }$ = 0.1 s^−^^1^; (**b**) $\dot{\varepsilon }$= 0.01 s^−^^1^; (**c**) $\dot{\varepsilon }$= 0.005 s^−^^1^.

Common methods for solving the dynamic recrystallization volume fraction of materials include metallographic method, energy method and true stress–strain curve method. However, the metallographic method has higher requirements on the metallographic cross section and corrosion results, and the energy method is inconvenient for measuring the deformation energy storage. Therefore, the true stress–strain curve method was selected to determine the dynamic recrystallization volume fraction of Cr8 alloy steel. The study by Laasraoui et al. [25] showed that the relationship between the DRX volume fraction $X_{drx}$ and stress of metallic materials during thermal deformation can be expressed as

(12) $$X_{drx}=\frac{\sigma_{drv}-\sigma }{\sigma_{sat}-\sigma_{ss}},$$

where $\sigma_{drv}$ represents the flow stress value that only considers the dynamic recovery, that is, the stress value that was obtained from the dynamic recovery curve of the material; $\sigma_{sat}$ represents the saturated stress of the DRV curve; $\sigma_{ss}$ represents the steady–state stress. According to the study by Poliak EI and Jonas J. J. [26], the saturated stress can be obtained by the θ−σ curve. Figure 7 is a typical θ−σ curve. The turning point represents the critical stress point, and the corresponding work−hardening rate was the critical work−hardening rate $\theta_{c}$. The θ−σ curves of Cr8 alloy steel are established as described in Figure 8. Therefore, the dynamic recovery curve under different conditions can be expressed by

(13) $$\begin{array}{cc} \sigma_{drv}=\sigma_{sat}+\left(\sigma_{c}-\sigma_{sat}\right)\exp\left(\frac{\left(\varepsilon -\varepsilon_{c}\right)\theta_{c}}{\sigma_{c}-\sigma_{sat}}\right) & \sigma_{c}\leq \sigma \leq \sigma_{sat} \end{array}.$$

**Figure 9:**
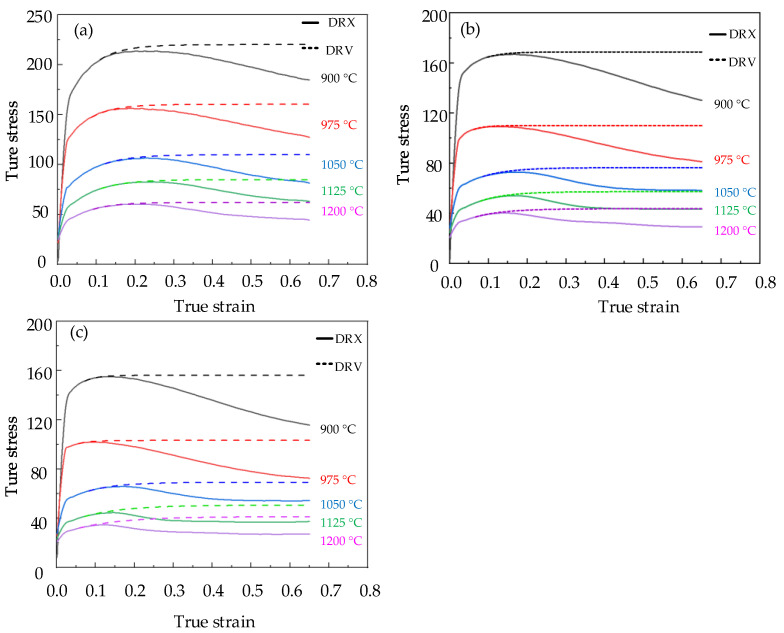
DRV curves and DRX curves of Cr8 alloy steel under different temperatures and strain rates: (**a**) $\dot{\varepsilon }$ = 0.1 s^−^^1^; (**b**) $\dot{\varepsilon }$= 0.01 s^−^^1^; (**c**) $\dot{\varepsilon }$= 0.005 s^−^^1^.

**Figure 10:**
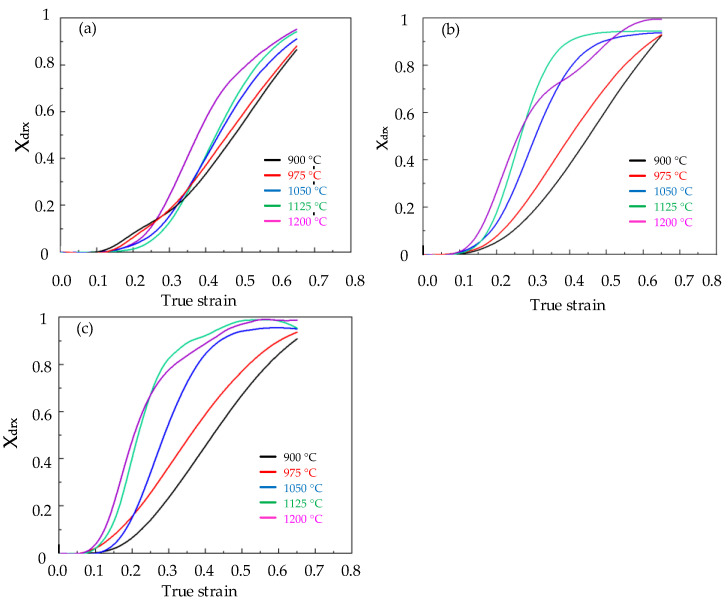
DRX volume fraction curve of Cr8 alloy steel under different temperatures and strain rates: (**a**) $\dot{\varepsilon }$ = 0.1 s^−^^1^; (**b**) $\dot{\varepsilon }$= 0.01 s^−^^1^; (**c**) $\dot{\varepsilon }$= 0.005 s^−^^1^.

Figure 8 shows the saturated steady state stress $\sigma_{sat}$, critical stress $\sigma_{c}$ and critical work−hardening rate $\theta_{c}$ of Cr8 alloy steel, and Table 4 contains the details. The dynamic recovery curves of Cr8 alloy steel were obtained by Formula (8), as shown in Figure 9. According to Formula (12) combined with Figure 9, the dynamic recrystallization volume fraction of Cr8 alloy steel was calculated as shown in Figure 10.

The characteristic strain $\varepsilon_{0.5}$ was the true strain corresponding to the dynamic recrystallization volume fraction reaching 50%. According to Figure 10, the characteristic strain $\varepsilon_{0.5}$ of the Cr8 alloy steel is described in Table 5.

The dynamic recrystallization volume fraction model proposed by Kopp et al. is depicted in

(14) $$\left\{ \begin{array}{l} X_{drx}=1-\exp\left(-k_{d}{\left(\frac{\varepsilon -\varepsilon_{c}}{\varepsilon_{0.5}-\varepsilon_{c}}\right)}^{n_{drx}}\right)\\ \varepsilon_{0.5}=A_{0.5}\cdot {\dot{\varepsilon }}^{m_{0.5}}\cdot \exp\left(\frac{Q_{0.5}}{RT}\right) \end{array}\right.,$$

where $X_{drx}$ represents the volume fraction of dynamic recrystallization, $\varepsilon_{0.5}$ represents the characteristic strain of Cr8 alloy steel under different conditions, $\varepsilon_{c}$ represents the critical strain of Cr8 alloy steel under different conditions, $k_{d}$, $n_{drx}$, $A_{0.5}$, $m_{0.5}$, $Q_{0.5}$ is a constant, where, when $\varepsilon =\varepsilon_{0.5}$, $X_{drx}$ = 0.5, then $k_{d}$ = ln2 = 0.693. Substitute $k_{d}$ = ln2 = 0.693 into Equation (14), and take the natural logarithm of the Formula to obtain

(15) $$\left\{ \begin{array}{l} \ln\left(-\ln\left(1-X_{drx}\right)\right)=\ln0.693+n_{drx}\cdot \ln\left(\frac{\varepsilon -\varepsilon_{c}}{\varepsilon_{0.5}-\varepsilon_{c}}\right)\\ \ln\varepsilon_{0.5}=\ln A_{0.5}+m_{0.5}\ln\dot{\varepsilon }+\frac{Q_{0.5}}{RT} \end{array}\right..$$

**Figure 11:**
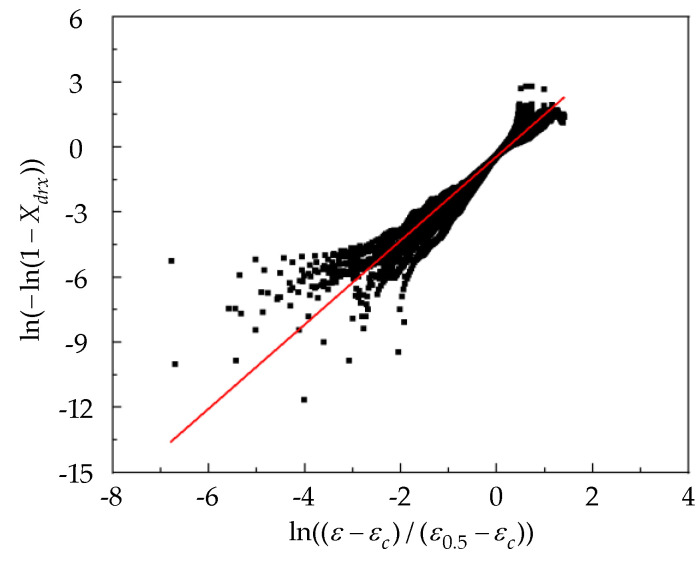
Relation curve of the $\ln((\varepsilon -\varepsilon_{c})/(\varepsilon_{0.5}-\varepsilon_{c}))$ and $\ln(-\ln(1-X_{drx}))$.

Therefore, a scatter plot of $\ln(\varepsilon -\varepsilon_{c})/(\varepsilon_{0.5}-\varepsilon_{c})-\ln(-\ln(1-X_{drx}))$ was established, and a linear fitted was performed to calculate $n_{drx}$= 1.93827, as shown in Figure 11.

To define the parameters of the characteristic strain model, when the constant strain rate was unchanged, $\ln(A_{0.5})+m_{0.5}\ln\dot{\varepsilon }$ was a constant, and the scatter plot of $\ln(\varepsilon_{0.5})-1/RT$ under different strain rates was established; subsequently, linear fit can be performed. The slope of the $\ln(\varepsilon_{0.5})-1/RT$ fitted curve was equal to the $Q_{0.5}$ value, and $Q_{0.5}$ = 33598.8945 J/mol. When the temperature was constant, $\ln(A_{0.5})+Q_{0.5}/RT$ was a constant, and a scatter plot of $\ln(\varepsilon_{0.5})-\ln\dot{\varepsilon }$ at different temperatures was established, and a linear fit was performed to find $m_{0.5}$ = 0.128768. The values of $m_{0.5}$ and $Q_{0.5}$ were substituted into Formula (15) to obtain the value of $A_{0.5}$ under different conditions, and the average value of $A_{0.5}$ = 0.02638 was obtained. Therefore, the dynamic recrystallization volume fraction model was

(16) $$\left\{ \begin{array}{l} X_{drx}=1-\exp\left(-0.693{\left(\frac{\varepsilon -\varepsilon_{c}}{\varepsilon_{0.5}-\varepsilon_{c}}\right)}^{1.93827}\right)\\ \varepsilon_{0.5}=0.02638\cdot {\dot{\varepsilon }}^{0.128768}\cdot \exp\left(\frac{33598.8945}{RT}\right) \end{array}\right..$$

**Figure 12:**
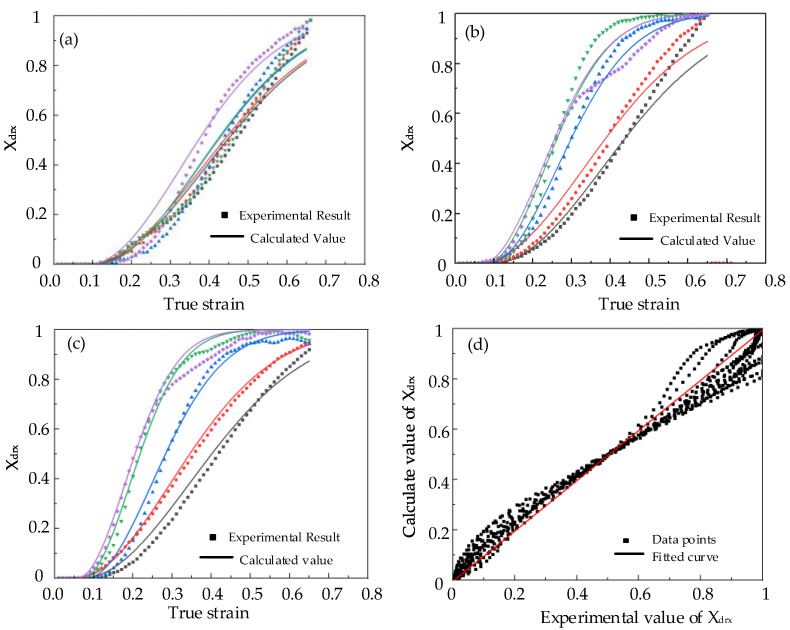
Comparison between calculated model and experimental result of DRX volume fraction: (**a**) $\dot{\varepsilon }$ = 0.1 s^−^^1^; (**b**) $\dot{\varepsilon }$ = 0.01 s^−^^1^; (**c**) $\dot{\varepsilon }$ = 0.005 s^−^^1^; (**d**) Comparison of calculated and experimental values.

The obtained DRX model and the experimental result are described in Figure 12. Figure 12a–c, respectively, show the experimental result and the calculated model of the DRX volume fraction of Cr8 alloy steel. The model curve agreed well with the experimental results. Figure 12d depicts the correlation study of the experimental value and the calculated value of the DRX volume fraction. The correlation coefficient R = 0.988 and the root mean square error RMSE = 0.053 were obtained by

(17) $$\begin{array}{l} R=\frac{\sum_{i=1}^{n}{\left(X_{e}^{i}-{\bar{X}}_{p}\right)}^{2}{\left(X_{p}^{i}-{\bar{X}}_{p}\right)}^{2}}{\sqrt{{\left(X_{e}^{i}-{\bar{X}}_{e}\right)}^{2}}\sqrt{{\left(X_{p}^{i}-{\bar{X}}_{p}\right)}^{2}}}\\ RMSE=\sqrt{\frac{1}{n}\sum_{i=1}^{n}{\left(X_{e}^{i}-X_{p}^{i}\right)}^{2}} \end{array},$$

indicating that it was effective to predict the DRX volume fraction of Cr8 alloy steel by the determined DRX model.

#### 3.2.3. Determination of Parameters for Grain Size Model

The metallographic sample of Cr8 alloy steel after hot compression was polished and corroded, and the experimental structure was observed by an Olympus–PMG3 optical microscope. The grain sizes $D_{drx}$ of Cr8 alloy steel were determined by the truncation method, as shown in Table 6. According to the literature, the grain size model was

(18) $$D_{\mathrm{drx}}=A_{D}\cdot d_{0}{}^{n_{D}}\cdot {\dot{\varepsilon }}^{m_{D}}\cdot \exp\left(\frac{Q_{D}}{RT}\right).$$

Because the role of initial grain size was not investigated, set $n_{0}$ = 0, and then, the grain size model could be expressed by

(19) $$D_{\mathrm{drx}}=A_{D}\cdot {\dot{\varepsilon }}^{m_{D}}\cdot \exp\left(\frac{Q_{D}}{RT}\right).$$

The $\ln(D_{drx})-1/RT$ curve and the $\ln(D_{drx})-\ln\dot{\varepsilon }$ curve were established by constant strain rate and temperature, respectively, and the grain size model of Cr8 alloy steel was obtained as

(20) $$D_{\mathrm{drx}}=56374.52371\times {\dot{\varepsilon }}^{-0.03113}\times \exp\left(\frac{-94442.26927}{RT}\right).$$

### 3.3. Analysis of Microscopic Evolution Based on Finite Element

Finite element method has been applied to the analysis of microstructural evolution during metal forming. The finite element simulation software can simulate and predict the microscopic evolution of metal in the forming process, such as DRX volume fraction, grain size, new grain shape nuclear, etc. [27,28,29].

**Figure 13:**
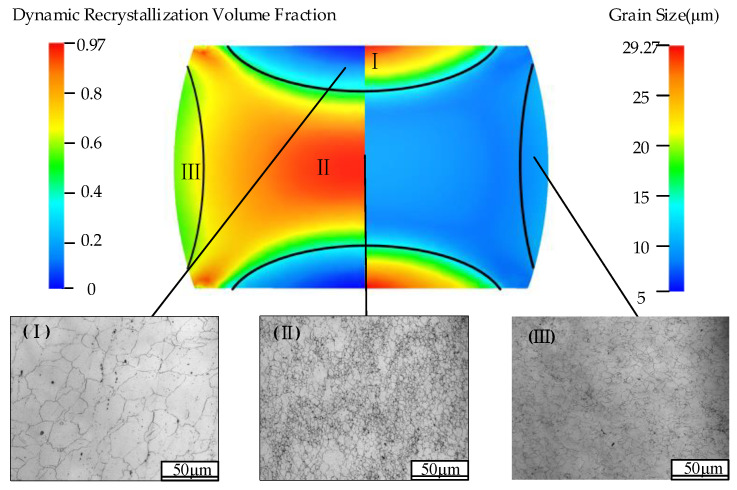
Comparison between simulation results and compression experiment at 1050 °C and 0.1 s^−1^: (**I**) difficult deformation region; (**II**) large deformation region; (**III**) small deformation region.

**Figure 14:**
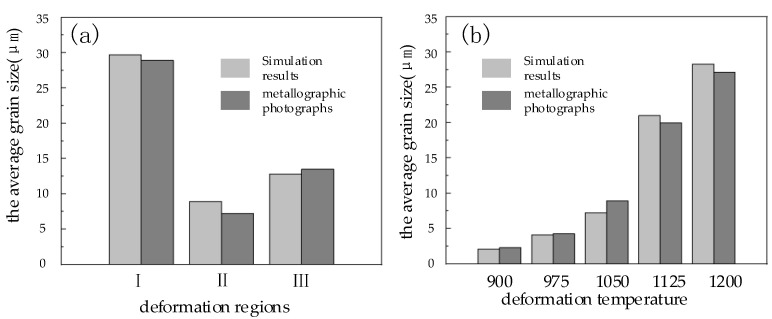
The comparison of the average grain size between simulation results and metallographic photographs: (**a**) The comparison of the average grain size under different deformation regions at 1050 °C and 0.1 s^−1^; (**b**) The comparison of the average grain size under different temperature at 0.1 s^−1^.

The compression process of Cr8 alloy steel cylindrical sample was simulated by the commercial simulation software Forge^®^ at 1050 °C and 0.1 s^−1^. The size of the cylindrical sample was ø 8 × 12 mm, and the compressive true strain was 0.65. The analysis result is shown in Figure 13. The upsetting specimen was divided into three deformation regions, which are: I: difficult deformation region near the both ends; II: large deformation region in the middle region; III: small deformation region near the sides of the cylinder. The left and right parts of the figure were the DRX volume fraction and average grain size of the Cr8 alloy steel after compression, and the metallographic photos of the three deformed regions of the sample after corrosion. The results state that in the difficult deformation area, the simulated DRX volume fraction was 0.014, the simulated average grain size equaled 29.68 μm, and the value of grain size determined by the corresponding metallographic photograph was 28.9 μm. In the large deformation area, the DRX volume fraction obtained by the simulation of Cr8 alloy steel was 0.947, the simulated average grain size equaled 8.92 μm, and the value of grain size determined by the corresponding metallographic photo was 7.2 μm; in the small deformation area, the DRX volume fraction obtained by the simulation of Cr8 alloy steel was 0.534, the simulated average grain size equaled 12.8 μm, and the value of grain size determined by the corresponding metallographic photograph was 13.5 μm. In addition, at 0.1 s^−1^ strain rate, the compression process of Cr8 alloy steel at 900 °C, 975 °C, 1125 °C and 1200 °C was simulated, and the average grain sizes of the large deformation regions were 2.28 μm, 4.26 μm, 19.95 μm and 27.14 μm, respectively. The average grain size obtained from the simulation results was compared with the grain size obtained from the metallographic photographs, the results are shown in Figure 14. Figure 14a is the comparison of the average grain size of three different deformation regions of the cylindrical specimen of Cr8 alloy steel at 0.1 s^−1^, 1050 °C; Figure 14b is a comparison of the average grain size at different temperatures at a strain rate of 0.1 s^−1^. By comparison, it is found that at 0.1 s^−1^ and 1050 °C, the relative error of the average grain size under different deformation regions obtained from the simulation results and the metallographic photos was 10.6%, and the relative error of the average grain size under different temperature was 9.1%, which showed that the simulation and experiment were congruent. The model can be used to predict the dynamic recrystallization structure evolution of Cr8 alloy steel, which provides theoretical guidance for optimizing roller forming process and refining grain.

## 4. Conclusions

As the key component of the rolling mill, the good microstructure and mechanical properties of the roller are the necessary aspect for the normal operation of the entire rolling production line. In order to effectively predict and control the microstructure and grain sizes inside the roller, this paper studied the dynamic recrystallization behavior and microstructure evolution of Cr8 alloy steel during hot forming based on the dynamic recrystallization model of Cr8 alloy steel and finite element software, which provided a reliable theoretical basis for optimizing the thermal processing parameters of the roller and manufacturing the roller with good structure and performance. The main conclusions are as follows:

The high temperature compression test of Cr8 alloy steel at 900–1200 °C, 0.005–0.1 s^−1^ was carried out by Gleeble−1500D thermal simulation compression testing machine, and the true stress–strain curve of Cr8 alloy steel at different temperatures and strain rates was obtained. The true stress–strain curve of Cr8 alloy steel exhibited typical dynamic recrystallization characteristics. The stress increased with the strain to the peak stress, and then gradually decreased and tended to be stable. This is caused by the combined action of work hardening, dynamic recrystallization and dynamic recovery when the material is deformed.

Based on the experimental data and Kopp dynamic recrystallization model, the dynamic recrystallization model of Cr8 alloy steel was established. In order to verify the accuracy of the model, the dynamic recrystallization volume fraction calculated by the model was compared with the dynamic recrystallization volume fraction obtained by the experiment. The correlation coefficient was 0.988, and the root mean square error (RMSE) was 0.053. It shows that the established model is basically consistent with the experiment.

In order to further verify the accuracy of the established dynamic recrystallization model of Cr8 alloy steel, through the secondary development of the finite element software Forge^®^, the established dynamic recrystallization model was embedded in the finite element software to simulate the compression test of Cr8 alloy steel under the condition of 0.1 s^−1^ and 1050 °C. The grain sizes of different deformation regions obtained by simulation were compared with the grain sizes obtained from the corresponding metallographic photos, and the average relative error of grain sizes was 10.6%. In addition, the cylindrical compression test of Cr8 alloy steel at different temperatures under the condition of 0.1 s^−1^ was simulated, and the grain sizes of the large deformation area of the Cr8 alloy steel cylindrical sample obtained by simulation at different temperatures and the grain sizes obtained by the corresponding metallographic photos were compared. The average relative error of grain sizes was 9.1%. The simulation results were in good agreement with the experimental results, indicating that the established model has high accuracy, which provides theoretical support for optimizing the thermal processing parameters of the Cr8 roller.

## Figures and Tables

**Table 1:** Chemical element compositions of Cr8 steel, wt%.

C	Si	Mn	Cr	Ni	Mo	V
0.48	0.54	0.60	7.46	0.48	0.45	0.13

**Table 2:** The values of steady state stress and peak stress of Cr8 alloy steel at 900–1200 °C and 0.005–0.1 s^−1^. (The units of strain rate are “s^−1^”, and the units of stress are “MPa”).

Strain Rate	σ_p_	σ_ss_	σ_p_	σ_ss_	σ_p_	σ_ss_	σ_p_	σ_ss_	σ_p_	σ_ss_
	900 °C		975 °C		1050 °C		1125 °C		1200 °C	
0.1	213.72	184.24	156.42	123.63	106.63	82.42	82.66	64.24	60.33	46.06
0.01	166.89	121.58	109.28	80.71	72.75	58.39	53.85	43.55	40.14	29.14
0.005	155.46	108.08	102.43	70.84	66.15	53.94	44.70	36.16	34.87	27.27

**Table 3:** Fitted parameters, critical strains and peak strains.

Strain Rate s^−1^	Temperature (°C)	Fitted Parameters				$\mathrm{Critical}\;\mathrm{Strain}\;\varepsilon_{c}$	Peak Strain $\varepsilon_{p}$ (MPa)
		A	B	C	D		
0.1	900	9.816	−108.498	1020.093	−3562.414	0.095	0.261
0.1	975	9.452	−119.361	1233.507	−4660.824	0.088	0.189
0.1	1050	8.243	−74.077	601.769	−1846.575	0.109	0.230
0.1	1125	7.737	−65.295	528.115	−1590.837	0.111	0.248
0.1	1200	7.465	−79.445	797.098	−2884.126	0.092	0.205
0.01	900	9.776	−115.985	920.360	−3099.343	0.099	0.189
0.01	975	9.659	−178.434	2372.403	−12,457.480	0.064	0.125
0.01	1050	8.261	−113.560	1203.309	−4599.237	0.087	0.174
0.01	1125	7.492	−97.478	1144.790	−4762.145	0.080	0.172
0.01	1200	7.276	−116.756	1630.287	−7899.406	0.069	0.139
0.005	900	10.011	−152.961	1723.502	−7913.370	0.073	0.163
0.005	975	9.578	−182.858	2332.164	−13,550.948	0.057	0.096
0.005	1050	8.242	−129.060	1515.713	−6121.176	0.083	0.159
0.005	1125	7.167	−97.242	1215.120	−5621.639	0.072	0.147
0.005	1200	6.820	−108.798	1681.708	−9461.163	0.059	0.137

**Table 4:** Saturated steady state stress $\sigma_{sat}$, critical stress $\sigma_{c}$ and critical work−hardening rate $\theta_{c}$ of Cr8 alloy steel.

Strain Rate (s^−1^)	Temperature (°C)	Saturated Steady State Stress (MPa)	Critical Stress (MPa)	Critical Work Harding Rate
0.1	900	220.30	205.39	241.66
0.1	975	160.42	147.68	213.976
0.1	1050	110.02	101.06	126.54
0.1	1125	84.97	76.05	129.28
0.1	1200	62.20	55.33	112.59
0.01	900	168.75	164.69	100.56
0.01	975	109.97	106.89	114.07
0.01	1050	76.36	69.33	98.80
0.01	1125	57.14	49.64	106.90
0.01	1200	43.54	37.07	85.00
0.005	900	156.25	151.66	146.88
0.005	975	103.33	100.8	76.00
0.005	1050	69.23	62.25	90.75
0.005	1125	50.74	41.56	87.18
0.005	1200	41.41	32.15	78.73

**Table 5:** Characteristic strain $\varepsilon_{0.5}$ of Cr8 alloy steel under different conditions.

ε_0.5_	0.1 s^−^^1^	0.01 s^−^^1^	0.005 s^−^^1^
900 °C	0.446	0.437	0.401
975 °C	0.438	0.387	0.345
1050 °C	0.422	0.299	0.283
1125 °C	0.420	0.262	0.218
1200 °C	0.372	0.256	0.205

**Table 6:** Grain size of Cr8 alloy steel under different conditions.

*D*_drx_ (μm)	0.1 s^−1^	0.01 s^−1^	0.005 s^−1^
900 °C	2.1	3.5	4.5
975 °C	4.1	7.4	6.9
1050 °C	7.2	20.6	16.4
1125 °C	21	15.2	14.9
1200 °C	28.3	26.1	33.1

## Data Availability

The data presented in this study are available on request from the corresponding author. The data are not publicly available due to these data are part of ongoing research.
